# Polymorphisms in Toll-like receptor genes influence antibody responses to cytomegalovirus glycoprotein B vaccine

**DOI:** 10.1186/1756-0500-5-140

**Published:** 2012-03-13

**Authors:** Ravit Arav-Boger, Genevieve L Wojcik, Priya Duggal, Roxann G Ingersoll, Terri Beaty, Robert F Pass, Robert H Yolken

**Affiliations:** 1 Department of Pediatrics, Division of Infectious Diseases, Johns Hopkins Hospital, Baltimore, Maryland 21287-4933, USA; 2 Department of Epidemiology Johns Hopkins Bloomberg School of Public Health, Baltimore, MD, 21231-1000, USA; 3 Department of Pediatrics, Division of Infectious Diseases, University of Alabama at Birmingham, Birmingham, Alabama 35294, USA

**Keywords:** Cytomegalovirus, Toll-like receptors, single nucleotide polymorphisms, glycoprotein B vaccine

## Abstract

**Background:**

Congenital Cytomegalovirus (CMV) infection is an important medical problem that has yet no current solution. A clinical trial of CMV glycoprotein B (gB) vaccine in young women showed promising efficacy. Improved understanding of the basis for prevention of CMV infection is essential for developing improved vaccines.

**Results:**

We genotyped 142 women previously vaccinated with three doses of CMV gB for single nucleotide polymorphisms (SNPs) in TLR 1-4, 6, 7, 9, and 10, and their associated intracellular signaling genes. SNPs in the platelet-derived growth factor receptor (PDGFRA) and integrins were also selected based on their role in binding gB. Specific SNPs in TLR7 and IKBKE (inhibitor of nuclear factor kappa-B kinase subunit epsilon) were associated with antibody responses to gB vaccine. Homozygous carriers of the minor allele at four SNPs in TLR7 showed higher vaccination-induced antibody responses to gB compared to heterozygotes or homozygotes for the common allele. SNP rs1953090 in IKBKE was associated with changes in antibody level from second to third dose of vaccine; homozygotes for the minor allele exhibited lower antibody responses while homozygotes for the major allele showed increased responses over time.

**Conclusions:**

These data contribute to our understanding of the immunogenetic mechanisms underlying variations in the immune response to CMV vaccine.

## Background

Infection with CMV is common in humans, causing severe morbidity and mortality in congenitally-infected newborns and in immunocompromised patients [1-3]. The importance of CMV as the leading infectious cause of mental retardation and deafness in children has been emphasized by its categorization by the Institute of Medicine as a level I vaccine candidate [4]. The rationale for developing a CMV vaccine is based on clinical and animal studies showing that immunity to CMV reduces the frequency and severity of disease [5,6]. In addition, animal studies demonstrated that immunization with subunit vaccines prevented disease and transplacental transmission of CMV [5-7]. Two recent phase II clinical trials with glycoprotein B (gB)-MF59 led to major enthusiasm and hope for the future success of CMV vaccine. The first was performed in young women recruited on postpartum wards [4], and showed 50% efficacy in preventing maternal CMV infection. Analysis of antibody levels to gB among vaccine recipients revealed that all women developed antibodies to gB although the levels and kinetics of antibody responses varied. The second study recruited patients from the kidney/liver transplant waiting list, and showed that antibody titers against gB were significantly increased one month after the second injection in patients given the vaccine compared with those given placebo, and that antibody titers to gB pretransplant correlated inversely with the duration of viremia, and the need for therapy with ganciclovir after transplant [8].

Data from human studies suggest that single nucleotide polymorphisms (SNPs) in immune response genes may influence severity of infections and response to vaccinations such as rubella, measles and hepatitis B [9-16]. Toll-like receptors (TLR) play a key role in the innate immune system and have been implicated in infectious and autoimmune processes [17]. CMV gB and glycoprotein gH (gH) associate with and activate TLR2/1, mediating an initial signal transduction pathway leading to upregulation of NF-kB and SP-1 [18,19]. In liver transplant recipients TLR2 R753Q SNP was associated with CMV replication and disease [20]. The successful gB vaccine trial in young women provided us with a unique opportunity to determine whether antibody responses to gB vaccine were influenced by SNPs in TLR genes.

## Methods

### Study population

The study cohort included healthy women who were enrolled in the CMV vaccine after obtaining written informed consent [4]. Women were screened on the postpartum wards, and those who were negative for antibody to CMV were invited to participate in the clinical trial. Study participants received a fixed dose of vaccine consisting of recombinant CMV envelope glycoprotein B (0.02 mg) with MF59 adjuvant (13.25 mg). The Johns Hopkins University School of Medicine and University of Alabama Institutional Review Boards granted approval for this study.

### Antibody assays

Antibody to CMV gB was measured using an Enzyme-linked immunoabsorbent assay (ELISA) [21]. The vaccine antigen, a recombinant gB molecule from Towne CMV (provided by Sanofi Pasteur, Marcy L'Etoile, France) was used.

### SNP selection

Using a candidate gene approach the following genes were selected: TLRs and associated intracellular signaling molecules: TLR1-4, TLR6, TLR7, TLR9, TLR10, JUN, MYD88, IKBKE, CHUK (IKKα), NF-KB1, CD14, MXD3 (MAD3), MAPK8 (JNK1), MAPK14, MAP3K7 (TAK1), LY96 (MD2), TRAF6, IRAK1, IRAK4, TBK1, TICAM1 (TRIF) and IRF3. In addition, PDGFRA, PDGFRB and integrin alpha V and integrin B1 were selected based on data showing their role in binding gB [22,23].

We identified tagging SNPs within the genes and in the 10 kb flanking each side of the genes, using the LDselect algorithm in individuals from the publicly available HapMap Yoruba, Nigeria population (YRI) [24]. For tagging SNPs we used an r^2 ^> 0.8. SNPs with a high r^2 ^are correlated and can give information about each other, so they are grouped together and one SNP is selected to represent them all. This is known as a "tagging" SNP. Selection was restricted to those SNPs with a minor allele frequency (MAF) > 1% in the African population. Additionally, all SNPs were evaluated using a proprietary algorithm from Illumina, Inc. (San Diego, CA) which assesses the likelihood that an assay will succeed on the Illumina genotyping platform. Only those with a design score > 0.8 were selected for use. For non-synonymous SNPs we used a more liberal cut off score > 0.4 to ensure inclusion of functional variants. Forty-one additional SNPs with previously reported associations in a variety of human diseases, 157 non-synoymous SNPs, and 28 ancestry informative markers (AIMs) were also typed for a total of 702 SNPs. African Americans are typically individuals with a mixed ethnic background. Allele frequencies of SNPs can vary by race and ethnicity. Therefore, it is important to assess the degree of mixture in an admixed individual when analyzing SNP data. If the cases and controls have differing amounts of admixture, variation in allele frequency by race can cause the appearance of an association with the phenotype of interest, when in reality the association is with race. Including AIMs in the SNP panel allows an assessment of admixture and means that differing amounts of admixture can be controlled for in the data analysis.

### Genotyping methods

Genomic DNA (samples, 75-150 ng/μl each) and 200-400 ng/μl (whole genome amplified DNA each) were obtained from frozen EDTA-blood samples using Gentra Puregene extraction (Qiagen). The study samples and duplicates were plated and genotyped together with 33 HapMap controls (21 CEU; 12 YRI). Genotyping was performed using the Illumina GoldenGate chemistry. Allele cluster definitions for each SNP were initially determined by Illumina's BeadStudio Genotyping Module (version 3.3.7) clustering algorithm using all samples in the project, applying a quality threshold (Gencall score) of 0.25. This was followed by manual review of the cluster definition for each SNP and adjustment of cluster boundaries to drop questionable genotype calls.

### Statistical analysis

28 ancestry informative markers were genotyped for evaluation of population stratification using principal components (PCA) in the statistical program Eigenstrat [25]. Six HapMap populations from Phase III (CEU, ASW, TSI, YRI, MKK and LWK) were also included. PCA was performed using the 28 AIMs and an additional 55 SNPs from the candidate genes without any linkage disequilibrium (LD). This second analysis did not increase the ability to discriminate between different ethnic groups, so only the results from the first analysis were incorporated into the regression as covariates, using the ten first principal components. Association analyses were done in PLINK, version 1.062. A principal component analysis (PCA) was applied to correct for potential confounding due to ancestry. A similar distribution of antibody levels was found among those of European and African ancestry (Additional file 1: Figure S1). In addition, quantile- quantile plots (QQ plots) including lambda values indicated that there was no increased stratification in our study population (Additional file 2: Figure S2). http://pngu.mgh.harvard.edu.ezproxy.welch.jhmi.edu/~purcell/plink/gplink.shtml using linear regression and an additive model. A Hardy-Weinberg p-value threshold of 10^-3 ^and a minor allele frequency (MAF) greater than 0.01 were used. A cut off at p value ≤ 0.03 was selected.

## Results

The study cohort was primarily African American (75%) with a median age of 19.6 years (range 14-40 years).

SNP data were available from 152 women who received gB vaccine (99% of attempted samples). Of 152 vaccine recipients, 142 received 3 doses of vaccine and had data available on both genotypes and ELISA titers. The titer of gB antibodies six month after administration of 3^rd ^vaccine ranged from 370-86,732 (median- 9847.7).

The first goal was to determine whether genetic variation within TLR genes contributed to the observed antibody responses to CMV gB vaccine. Linear regression was performed of individuals' log transformed ELISA levels measured 6 months after the third dose of vaccine. SNPs yielding p-value < 0.03 are summarized in Table 1. Four SNPs in TLR7 (rs179008, rs179009, rs179013 and rs179018) appeared to influence the level of antibodies to gB (Table 1). The linear regression model included potential confounding effects of age, race, educational level, and number of children. Results were similar without adjusting for these covariates (data not shown).

**Table 1:** Associations between SNPs in TLRs and gB-Specific Antibody Responses After 3 doses of CMV vaccine

Gene	SNP	Location	Minor Allele	Counts*	β	Standard Error	P value	Median ELISA**
*TLR7*	rs179009	intron	*C*	4/47/91	0.386	0.116	0.001	21102/13194/9769
*TLR7*	rs179008	coding	*T*	4/34/104	0.304	0.128	0.019	21102/12907/10340
*TLR7*	rs179018	intron	*G*	4/36/102	0.318	0.130	0.016	16610/12907/10691
*TLR7*	rs179013	intron	*T*	3/34/105	0.315	0.136	0.022	22940/13501/10530
*IKBKE*	rs1953090	intron	*C*	15/62/64	-0.275	0.097	0.005	10904/9676/14993
*TLR3*	rs3775292	intron	*G*	5/40/96	0.296	0.117	0.012	10904/13685/9662
*IRAK4*	rs1838341	intron	*G*	0/16/126	-0.458	0.204	0.027	NA/6394/11378
*TLR9*	rs5743849	intron	*T*	0/16/126	-0.469	0.211	0.028	NA/6635/11424

*Counts are presented as the number of women homozygous for the minor allele/heterozygous/homozygous for the major allele

**Median ELISA- the median gB antibody titer measured in each of the three groups

**Figure 1:**
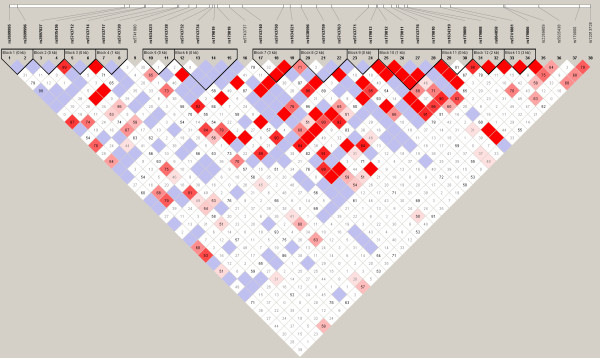
**LD Plot of SNPs in TLR7**. The blocks are defined by the black triangles using a solid spine of LD in Haploview. The color gradient refers to the degree of linkage disequilibrium, with red indicating strong LD (D' is high) and white indicating low LD (D' is low). Blue coloring indicates less confidence in the measurement. The actual LD is indicated in the box on a scale of 0-1.

Homozygous carriers of the minor allele at four SNPs in the TLR7 gene showed higher vaccination-induced antibody responses to gB compared to heterozygotes or homozygotes for the common TLR7 allele (Table 1). Three of these four SNPs (rs179009, rs179013, and rs179018) are located in the intronic regions. Rs179008 and rs1790099 are in strong linkage disequilibrium (Figure 1).

Next, a possible association of SNPs in TLRs with changes in antibody responses from one month after the second dose of vaccine to six months following the third (and last) dose was investigated. For this analysis, we used data from 69 women who initially were at the top 50% of responses (a cut off of 9,506.5 ELISA units was used), and then the change in antibody levels following the last dose of vaccine. Data were compared between women who had no change or an increase in antibody levels, versus those who had a decrease in antibody level. The OR was calculated as the odds of being a weak responder (i.e. having a negative change between visits) versus a strong responder (i.e. having no or positive change) in those who carried the risk allele versus not (Table 2). Two other genes with suggestive association include integrin αV and MAPK8. Rs1953090 appears to affect changes in antibody level with subsequent immunization with homozygous for the minor allele exhibiting lower antibody levels while homozygotes for the major allele showing increased responses.

**Table 2:** Associations between SNPs in TLRs and Changes in Antibody Responses between Second and Third Dose of CMV Vaccine

GENE	SNP ID	Location	Genotype Counts	Fisher's p- value	Median ELISA
*IKBKE*	rs1953090	intron	7/30/31	0.0009319	-14103/-2881/3047
*ITGAV*	rs3795865	intron	1/27/41	0.0123	-36266/-3084/1821
*MAPK8*	rs17010454	intron	0/8/61	0.02247	0/2862/-1708

## Discussion

Our analysis suggests that polymorphism in TLR genes may influence antibody responses to CMV gB vaccine. Specifically, SNPs in TLR7 and IKBKE may influence these responses. Viral nucleic acids are a major class of pathogen-associated molecular patterns recognized by TLRs. TLR3 (dsRNA), TLR7 (ssRNA), TLR8 (ssRNA), and TLR9 (CpG DNA) signal from the endosome where degradation of virus particles exposes the viral genome. Several adapter proteins interact with the TLRs: MyD88 (TLR1, 2, 5, 6, 7, 8, and 9), Mal (or Tirap) (TLR2 and 4) and TRIF (TLR3). These adapters link TLR proteins to a downstream signaling pathway including IRAK-1, IRAK-2, TRAF6, and IKK- αβγ. The primary consequences of TLR activation include NF-κB activation, inflammatory cytokine secretion, up-regulation of immune co-stimulatory molecules and, for a subset of TLRs, the production of type I interferon.

Herpesviruses activate signaling through TLR2 and TLR9 [26-28]. TLRs 2, 3, and 9 are involved in innate immune responses to mouse CMV infection [29,30]. Studies of human CMV have shown that both gB and gH activate TLR2 and physically associate with TLR2 and TLR1 [18]. TLR2 and CD14 recognize CMV virions, and cells exposed to soluble forms of gB activate NF-kB and type I interferon [31-33]. CMV gB and gH mediate an initial signal transduction pathway leading to upregulation of NF-kB and SP-1, an effect which is inhibited by neutralizing antibodies to gB and gH [19]. A specific single-nucleotide polymorphism (SNP) in TLR2 was associated with CMV replication and disease in a cohort of 92 liver transplant recipients [20].

Surprisingly, we found that specific SNPs in TLR7 were associated with variability in gB antibodies. SNP rs179008 is associated with amino acid change in position 11 of the TLR7 protein from glutamine to leucine. Several recent studies provide support for a functional effect of this SNP [34,35]. TLR7 is generally considered to function as an intracellular receptor for viral RNAs, but recent data suggest TLR7 exhibits a much broader range of specificities and can interact with endogenous RNAs, as well as bind directly to bacterial pathogens [36,37]. Among viruses, Epstein Barr virus (EBV), a member of the Herpesvirus family, interacts with the TLR7 pathway and enhances B cell proliferation [38]. The role of TLR7 in the immune response to CMV has been suggested in a study that used plasmacytoid dendritic cells (PDCs), the main producers of type I IFN in response to viral infection. Although PDCs were not permissive to CMV infection, they secreted cytokines after contact with CMV, including IFN-α secretion that was blocked by inhibition of TLR7 and TLR9, suggesting an engagement of the TLR7 and/or TLR9 pathways. Through PDC stimulation, CMV prompted B cell activation, but only induced antibody production in the presence of T cells [39].

The best known TLR member expressed in human B cells is TLR9 [40], but other TLRs including TLR7 are also expressed. TLR7 ligands activate murine B cells [41] but TLR7 responsiveness of naive human B cells is controlled by PDC [42]. IFN-α released from PDCs upon stimulation with TLR7 or TLR9 ligands was found to be responsible for increased TLR7 sensitivity of B cells. Depletion of PDC from mixed cell cultures (PBMC) lead to a complete loss of TLR7 ligand-induced B cell proliferation. Our data suggest that TLR7 could interact with one of the components of the gB vaccine, or that vaccination induces upregulation of endogenous RNAs subsequently recognized by TLR7. Activation of TLR7 upon gB immunization may induce B-cells to proliferate and generate antibodies. While the MF59 adjuvant could also play a role in TLR7 activation, a recent study showed that MF59 did not activate any of the TLRs in vitro [43]. We note that activation via TLR pathways is not essential for the function of every adjuvant [44]. Alum adjuvant, as well as complete and incomplete Freund's adjuvants, enhanced antibody responses in mice genetically-engineered to be unable to signal through the major TLR adapters MyD88.

IKBKE, a noncanonical I-kappa-B kinase (IKK), is essential for regulating antiviral signaling pathways. It phosphorylates inhibitors of NF-kappa-B, leading to dissociation of the inhibitor/NF-kappa-B complex and ultimately degradation of the inhibitor. IKBKE is expressed predominantly in immune cells and tissues, including peripheral blood leukocytes, spleen, and thymus [45]. IKBKE phosphorylates IRF-3 and is required for its full activation in CMV-infected smooth muscle cells [46]. Thus, it has a pivotal role in coordinating the activation of IRF3 and NF-kB in the innate immune response. Although IRF-3 was initially reported to be activated by recombinant gB [47], recently Z-DNA binding protein 1, ZBP1 was suggested as an essential gene for IRF3 activation by CMV [48]. A recent study demonstrated that induction of inflammatory cytokines by CMV was mediated via a TLR-2-dependent activation of NF-κB [31], but it did not specifically evaluate whether IRF-3 was activated in a TLR-2-dependent fashion by CMV. So far, the literature has only shown TLR-3 and TLR-4 can induce type I interferon production through IRF-3 activation. The ability of TLR-7 to induce type I interferon through IRF-3 has not been studied yet.

## Conclusions

This study shows for the first time that host genetics plays a role in the development of gB antibodies following CMV immunization. It is unclear whether these polymorphisms contribute to protective immunity. Future studies will provide additional understanding of the roles of immune response genes in CMV immunization and will evaluate the role of gB in activation of TLR7-related pathways.

### Availability of supporting data

The data set supporting the results of this article is included within the article

## Abbreviations

CMV: Cytomegalovirus; gB: glycoprotein B; TLR: Toll-like receptors; PDGFR: Platelet derived growth factor receptor; IKBKE: Inhibitor of nuclear factor kappa-B kinase subunit epsilon; SNP: Single nucleotide polymorphism

## Authors' contributions

RAB conceived of the study, its design and wrote the manuscript; GLW, PD and TB performed and reviewed the statistical analysis, RGI helped in SNP selection, RFP provided essential samples and edited the manuscript, RHY provided input into experimental design. All authors read and approved the final manuscript.

## Competing interests

Ravit Arav-Boger received research funding from Sanofi-Pasteur. Robert F Pass receives research funding from Sanofi-Pasteur. In addition, Dr. Pass has served as a consultant to Merck, Vical, AlphaVax and MedImmune. Other authors: no conflict.

## Supplementary Material

Additional file 1 **Figure S1**. SNPs in ancestry informative markers and antibody levels in the study population. A principal component analysis was performed to correct for potential confounding due to ancestry. The distribution of antibody levels is depicted for African and European women. Click here for file

Additional file 2 **Figure S2**. Quantile-Quantile (QQ) plots of the log antibody levels QQ plots of log antibody levels and lambda values are depicted. In the right panel, nothing is done to account for the structure. On the left panel, the results are adjusted for principal components, leaving about the same amount of inflation as the case with no population stratification. Click here for file
