# Supplementary figures and images for: Polymorphisms in Toll-like receptor genes influence antibody responses to cytomegalovirus glycoprotein B vaccine
Source: BMC Res Notes. 2012 Mar 13;5:140. doi: 10.1186/1756-0500-5-140 (PMC3317442; doi:10.1186/1756-0500-5-140)

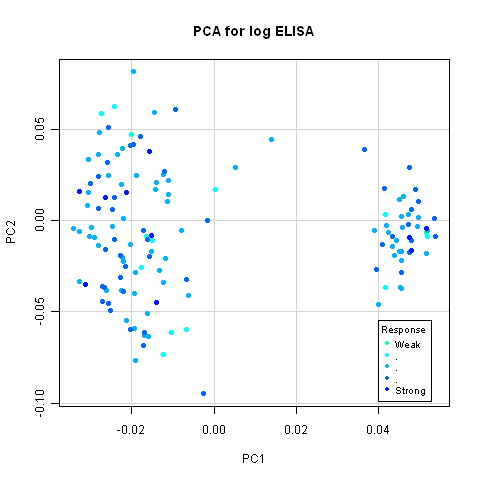

Supplement: Additional file 1 — Figure S1. SNPs in ancestry informative markers and antibody levels in the study population. A principal component analysis was performed to correct for potential confounding due to ancestry. The distribution of antibody levels is depicted for African and European women. [file 1756-0500-5-140-S1.PNG]

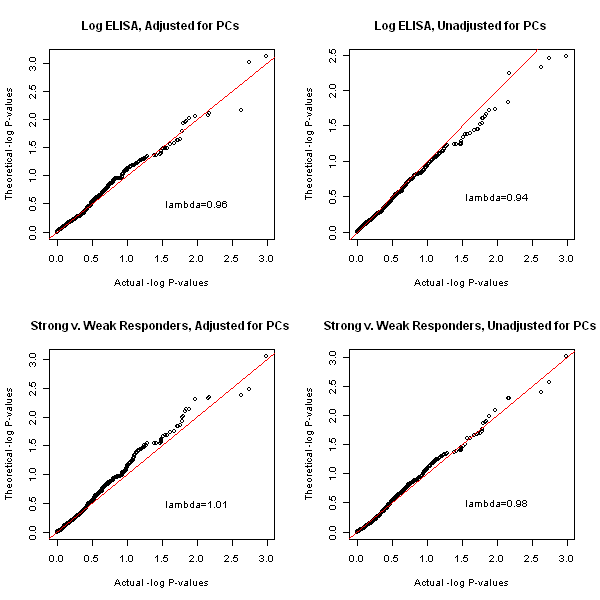

Supplement: Additional file 2 — Figure S2. Quantile-Quantile (QQ) plots of the log antibody levels QQ plots of log antibody levels and lambda values are depicted. In the right panel, nothing is done to account for the structure. On the left panel, the results are adjusted for principal components, leaving about the same amount of inflation as the case with no population stratification. [file 1756-0500-5-140-S2.PNG]
